# Evaluating the rumen microbial community of genetically divergent spring-calving dairy cows grazing grass-only or grass-clover swards at different stages of the grazing season

**DOI:** 10.3389/fmicb.2025.1642486

**Published:** 2025-11-06

**Authors:** Charles Dwan, Anubhav Das, Paul W. O’Toole, Tom F. O’Callaghan, Dara Meehan, Deirdre Hennessy, Hannah Irish, Frank Buckley, Ben Lahart

**Affiliations:** 1 Teagasc, Animal and Grassland Research and Innovation Centre, Moorepark, Fermoy, Ireland; 2 School of Biological, Earth and Environmental Sciences, University College Cork, Cork, Ireland; 3 School of Microbiology & APC Microbiome Ireland, University College Cork, Cork, Ireland; 4 School of Food and Nutritional Sciences, University College Cork, Cork, Ireland

**Keywords:** dairy, rumen microbiome, pasture, season, enteric methane, clover, breeding, rumen fermentation characteristics

## Abstract

The current study used a culture-independent methodology to investigate the rumen microbiome composition in two genetically divergent groups of spring-calving dairy cows, high (€218) and low (€157) Economic Breeding Index (EBI), grazing two sward treatments—perennial ryegrass (grass-only) or perennial ryegrass and white clover (grass-clover)—at three time points across spring, summer, and autumn of a single grazing season. The analysis indicated that the EBI status had no significant effect on the rumen microbial community within the statistical power of this study. Beta diversity between the microbiomes was different (*p* < 0.001) between the two sward treatments only in autumn, when the clover proportion was highest (50.2%). Season had a significant effect on microbiome beta diversity across sward treatments (*p* < 0.001). There were only minor differences in the composition of the rumen microbiomes between the two sward treatments. Many bacterial genera were differentially abundant between spring and the two later time points. Bacterial genera that were more abundant in spring were positively correlated with rumen propionate levels, while those more abundant in summer and autumn were negatively correlated with propionate and positively correlated with acetate and butyrate. Methanogenic archaeal abundance was greater in summer and autumn compared to spring, and they were negatively correlated with propionate and positively correlated with methane (CH_4_) production. The results of this study demonstrate that genetic selection using the EBI does not affect the rumen microbial community and the core rumen microbial community is similar in cows grazing either grass-only or grass-clover swards. The results also demonstrate that the rumen bacterial community shifts across the grazing season, providing more favorable conditions for methanogenesis in summer and autumn compared to spring.

## 1 Introduction

The rumen of dairy cows has a complex and diverse microbial ecosystem that plays an important role in the digestion of plant material from which energy is provided to its host (Van Soest, 1994). This symbiotic relationship is essential for the production of nutrient-dense milk for human consumption (Mottet et al., 2018). However, hydrogen (H_2_) is a by-product of rumen fermentation that is removed to maintain redox balance and efficient fermentation in the rumen (Janssen, 2010). Hydrogen removal is achieved primarily by methanogenic archaea, which utilize excess H_2_ in the reduction of carbon dioxide (CO_2_) to methane (CH_4_), the majority of which is ultimately eructated by the animal into the atmosphere (Tapio et al., 2017). Enteric CH_4_ is a potent greenhouse gas (GHG) and a major contributor to agricultural emissions (IPCC 2019).

Due to the fact that microbial populations associate with different substrates, diet has a major influence on the rumen microbiota of their host (Henderson et al., 2015). In temperate regions with consistent annual grass growth, the diet of dairy cows largely consists of grazed pasture (Macdonald et al., 2001; O’Brien et al., 2018). In recent years, the inclusion of legumes in pasture-based systems, such as white clover (*Trifolium repens* L.; clover), has gained increasing importance, as their nitrogen (N) fixation ability offers the potential to offset the amount of artificial N fertilizer required (Murray et al., 2023). In addition, grass-clover swards have improved feed quality compared to grass-only swards, increasing dry matter intake (DMI), which often leads to increased animal performance (Dineen et al., 2018; Egan et al., 2018). However, the increased DMI associated with clover has been reported to increase enteric CH_4_ emissions (Lee et al., 2004; Dwan et al., 2025), although some studies have reported that clover reduces CH_4_ yield (CH_4_ per unit of DMI; Lee et al., 2004; Enriquez-Hidalgo et al., 2014). Previous studies have reported subtle differences in the rumen microbial communities of animals grazing grass-only and grass-clover swards (Smith et al., 2020; Woodmartin et al., 2024). Furthermore, grass-only and grass-clover swards both vary in quality across the grazing season (Hearn et al., 2022). Previous studies have also noted variation in CH_4_ emissions from dairy cows over the grazing season, the cause of which has been postulated to be partly due to shifts in pasture quality (Robertson and Waghorn, 2002; Lahart et al., 2024). The rumen microbial community of cows grazing grass-clover swards has been previously shown to undergo minor alterations over the grazing season with shifting sward quality and changes in lactation stage (Noel et al., 2017). However, these alterations have not been characterized in grass-only swards or with a focus on methanogenesis. To better understand seasonal variation in the CH_4_ yield of pasture-based dairy systems, the dynamics of the rumen microbial community structure across the grazing season in both grass-only and grass-clover swards must be further investigated.

In addition to diet, there is some evidence for host influences on the rumen microbial community, whereby differences have been observed between dairy cow breeds (King et al., 2011; Noel et al., 2019; Olijhoek et al., 2022) and between production, efficiency, and CH_4_ phenotypes within breeds (Tong et al., 2018; Lopes et al., 2021; Zhu et al., 2021; Stepanchenko et al., 2023). However, differences in microbiome composition between phenotypes are not always consistent, nor are they necessarily genetically driven (Fregulia et al., 2024). Previous research has shown that dairy cows ranking higher on the Irish total merit index, the Economic Breeding Index (EBI; www.icbf.com), have greater milk solids (milk fat + protein) production and/or greater feed efficiency (milk solids per kg of DMI) (O’Sullivan et al., 2019; Lahart et al., 2024; Dwan et al., 2025). The EBI is a total merit index that combines various genetic traits and subindexes into a single monetary value that represents an animal’s potential profitability within the context of Irish pasture-based dairy systems (Veerkamp et al., 2002). Research has demonstrated that improvements in performance associated with the EBI are not associated with greater CH_4_ emissions. However, there is currently limited information on whether selected genetic differences can influence the rumen microbial community, particularly in the context of enteric CH_4_ emissions.

Therefore, this study aimed to compare the rumen bacterial and methanogenic archaeal communities of high EBI and low EBI spring-calving Holstein Friesian dairy cows grazing grass-only or grass-clover swards at different stages of the grazing season.

## 2 Materials and methods

### 2.1 Experiment design

All experimental procedures conducted on the animals in the study were approved by the Health Products and Regulatory Authority (Dublin, Ireland) under project authorization AE19132/P138. The reported experiment was undertaken in 2022 at the Teagasc, Animal and Grassland Research and Innovation Centre, Moorepark, Fermoy, Co. Cork, Ireland. This study was part of a larger farm systems study described by Dwan et al. (2025), which involved two Holstein Friesian dairy cow genotypes of high and low genetic merit (€218 and €157 EBI, respectively). The cows in each EBI group were distributed evenly across two sward treatments: perennial ryegrass (*Lolium perenne* L.; PRG) receiving 225 kg artificial N/ha/yr (grass-only) and PRG with white clover (*Trifolium repens* L.) receiving 150 kg artificial N/ha/yr (grass-clover). The experiment consisted of three 14-day measurement periods: spring (27th of April to the 10th of May), summer (6th of July to the 19th of July), and autumn (21 September to 4 October). The study parameters comprised 28 spring-calving Holstein-Friesian dairy cows with average “days in milk” values of 70 in spring, 140 in summer, and 217 in autumn (SD = 15.6). A total of 14 cows were randomly selected from each sward treatment, balanced for EBI group, parity, milk production, milk composition, body weight, and body condition score. The cows were managed in a rotational grazing system similar to that described by Roche et al. (2017). The target pre-grazing herbage mass (>4 cm) was 1,300–1,600 kg DM/ha, and daily herbage allocations (>4 cm) were approximately 17 kg DM/cow/day. Residency time in each allocation was determined by a target post-grazing sward height of 4.0 to 4.5 cm measured using a rising plate meter (Jenquip Ltd.; Fielding, New Zealand).

### 2.2 Animal measurements

Animal measurements have been previously outlined by Dwan et al. (2025). In brief, CH_4_ emissions were measured using two Greenfeed units (C-lock Inc.; South Dakota, USA), which were swapped between the treatments twice during each measurement period. The cows received a target concentrate supplementation of approximately 1 kg/cow from the Greenfeed units per day. Milk production was measured daily, and milk fat and protein content were measured from subsequent AM and PM milk samples collected twice during each measurement period. Dry matter intake was estimated using the n-alkane method described by Dillon and Stakelum (1989) from days 6 to 10 during each measurement period. All measurements were then averaged per day across the measurement period.

### 2.3 Rumen fluid

Rumen fluid samples were collected from each animal on the final day of each measurement period using a transesophageal sampling device (FLORA rumen scoop; Guelph, ON, Canada). Rumen fluid was passed through cheesecloth, and 8 mL was added to 2 mL of 50% trichloroacetic acid. The samples were then stored at −18 °C until analysis. The remaining non-acidified sample was stored separately at −80 °C. The acidified samples were analyzed for volatile fatty acids (VFAs; acetate, propionate, butyrate, valerate, isobutyrate, isovalerate) and ammonia concentration, as previously described by Dwan et al. (2025). Insufficient fluid was collected from one cow; therefore, it was excluded from the analysis. Three cows were also excluded due to health reasons not related to the experiment, and an additional two were excluded as they had insufficient methane data. A breakdown of the number of subjects used in the final analysis is presented in Table 1.

**Table 1:** The number of subjects (n) analyzed within each breed group and sward treatment across the three measurement periods.

	GO		GC		Total
	High EBI	Low EBI	High EBI	Low EBI	
Spring	6	7	5	4	22
Summer	6	7	5	4	22
Autumn	6	7	5	4	22
Total	18	21	15	12	66

GO, grass-only; GC, grass-clover; EBI, economic breeding index.

### 2.4 DNA extraction, library preparation, and sequencing

DNA was extracted from 250 mg of the non-acidified rumen fluid samples using the repeated bead beating and column purification method described by Yu and Morrison (2004). DNA quality was assessed on agarose gels, and DNA concentration was quantified using a Nanodrop 1,000 spectrophotometer. The DNA samples were then standardized to a volume of 25 μL at a concentration of 20 ng/μl of DNA per sample. They were then shipped on ice to Novogene (UK) Co., Ltd. for PCR and sequencing.

Amplicons targeting the V4 region of the 16S rRNA gene were generated using the 515F/806R primers with Illumina technology. Sequencing libraries were generated, and indexes were added. The libraries were checked using Qubit and real-time PCR for quantification and a bioanalyzer for size distribution detection. The quantified libraries were pooled and sequenced on Illumina platforms, according to effective library concentration and the required data amount. Paired-end reads were merged using FLASH (Magoč and Salzberg, 2011). Quality filtering on the raw tags was performed using fastp (Bokulich and Mills, 2012). The SILVA database^1^ was used as a reference to detect chimeric sequences. The chimeric sequences were removed using the vsearch package (Edgar et al., 2011). Effective tags were cleaned using DADA2 to obtain initial amplicon sequence variants (ASVs). Taxonomic annotation was performed using QIIME 2 software, primarily with the SILVA database. For sequences that could not be annotated using SILVA, NCBI BLAST analysis was used to supplement the taxonomic information. The ASVs were separated into their individual kingdoms (Bacteria and Archaea). The archaeal ASVs were further classified against the RefSeq database^2^ using DADA2 in R to determine the proportion belonging to the *Methanobrevibacter* SGMT (smithii, gottschalkii, millerae, and thaueri) and RO (ruminantium and olleyae) clades, as defined by St-Pierre et al. (2015). Separate ASV tables were then constructed for the bacteria and archaea.

### 2.5 Data and statistical analysis

To investigate alpha diversity, the bacterial and archaeal ASV tables were each converted into phyloseq objects in R using phyloseq (McMurdie and Holmes, 2013). Each was then rarefied to its respective minimum sequencing depth (43,009 and 604 reads, respectively), following which observed ASVs and Shannon diversity were calculated for each sample’s respective bacterial and archaeal populations. Due to poor species-level classification, bacterial and archaeal population composition was aggregated to the genus level and the clade level in the case of *Methanobrevibacter*. Principal components and the Procrustes test were used to check if the aggregated data were similar in structure with the original data (*p* < 0.001) using the vegan R package (Oksanen et al., 2007). The aggregated tables were converted into phyloseq objects and rarefied to their respective minimum read depths after aggregation (35,502 and 604 reads for bacteria and archaea, respectively).

#### 2.5.1 Bacterial alpha and beta diversity

Bacterial alpha diversity was compared between the genotypes, the sward treatments and seasons using the Kruskal–Wallis test, followed by pairwise comparisons with the Dunn test adjusted using the Benjamini–Hochberg (BH) method. Only bacterial genera present in >5% of the samples were included in the beta diversity analysis. Principal coordinates analysis (PCoA) based on the Bray–Curtis dissimilarity was performed to visualize differences in bacterial beta diversity at the genus level. Co-inertia plots were generated, displaying BH-adjusted significant Spearman correlations between bacterial genera and the principal axes. A plot showing significant correlations (*p* < 0.05) of animal performance, gaseous emissions, rumen fermentation characteristics, and experimental factors with the PCoA ordination was generated using the envfit function from the vegan package in R. The experimental factors tested were genotype, sward treatment, season, and parity. Statistical differences in beta diversity were identified using PERMANOVA with the adonis2 function from the vegan package in R.

#### 2.5.2 Differential abundance testing

The analysis of composition of microbiomes (ANCOM) test was carried out on the non-rarefied bacterial ASVs present in >5% of the samples. Differences were identified at the genus level between the genotypes, sward treatments, and seasons using the ANCOM-BC package in R with a BH adjustment (Lin and Peddada, 2024).

#### 2.5.3 Archaeal population

The ratio of archaeal to bacterial reads, archaeal alpha diversity, and the relative abundance of the archaeal genera and clades were analyzed using the MIXED procedure in SAS, with genotype, sward treatment, measurement period, and their interactions as fixed effects (Woodmartin et al., 2024). A linear mixed model was used, as all archaeal data were normally distributed and taxonomic diversity was low both at the genus (two genera) and clade levels (two clades). Individual animal was included as a random effect, with measurement period as a repeated measure and a compound symmetry correlation structure. Other effects tested were parity and calving day of the year, as well as all possible interactions, but none of which were included in the final model as they were not significant for any variable tested (*p* > 0.05). The results were expressed as least square means, and the multiple comparisons were adjusted using the Tukey–Kramer method.

#### 2.5.4 Partial correlations

Spearman partial correlations, adjusted for genotype, sward treatment, measurement period, and parity, were used to investigate the relationship between microbial taxa and CH_4_ emissions, animal performance, and VFA concentrations using R.

## 3 Results

Grazing results, herbage chemical composition, enteric emissions, animal performance, and rumen VFA results have been reported previously by Dwan et al. (2025). A total of 6,386,864 reads were obtained from the 16S amplicon sequencing analysis, with a mean (standard deviation) number of reads per sample of 96,770 (26,160.3). Following quality filtering, merging, and removal of chimeric sequences, there was a total of 6,103,700 reads, with a mean of 92,480 (24,979.7) reads per sample. These reads were mapped to 8,510 ASVs, with a mean of 1,292 (340.8) ASVs per sample.

### 3.1 Rumen bacterial microbiome analysis

**Figure 1:**
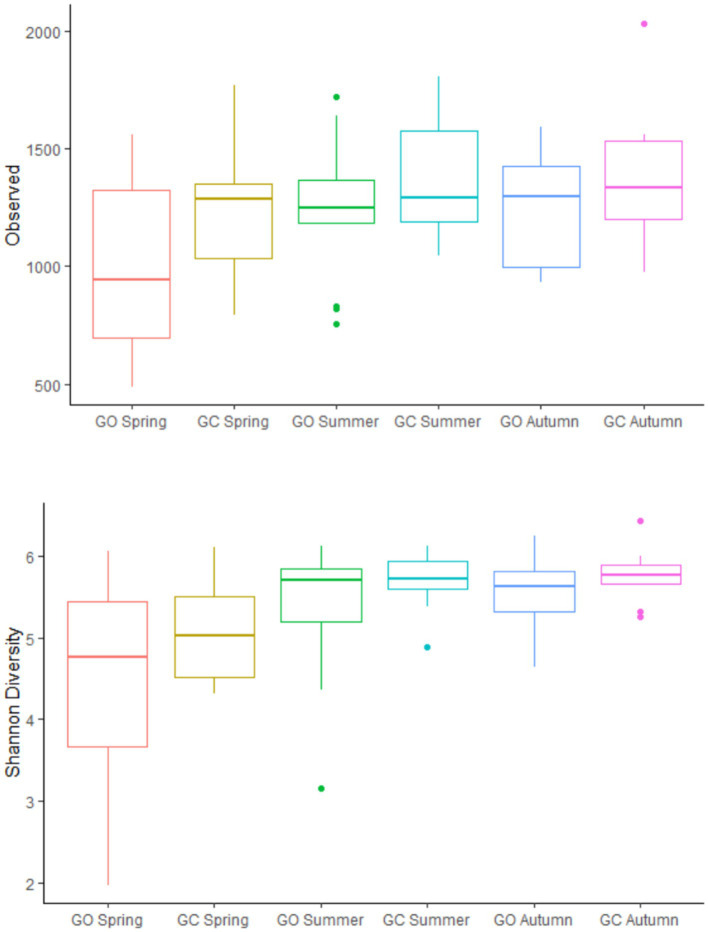
Observed ASVs and Shannon diversity of the rumen bacterial populations of cows grazing grass-only swards (GO; *n* = 13) and grass-clover swards (GC; *n* = 9) in spring, summer, and autumn.

Across both sward treatments and all measurement periods, *Prevotella* (32.2%) was the most prevalent bacterial genus, followed by *Olsenella* (11.0%), the *Christensenellaceae R7 group* (9.49%), and *Kandleria* (6.62%; Supplementary Figure 1). On average, *Erysipelotrichaceae UCG 002* was also a dominant genus (5.52%); however, this genus was more prevalent in grass-only swards during spring (20.3%) and was skewed by two samples with particularly high abundances (78.5 and 58.7%, respectively). There was no effect of the EBI group on the bacterial alpha diversity. The effects of sward treatment and measurement period on bacterial alpha-diversity are presented in Figure 1. Grass-clover swards had a greater (*p* < 0.05) number of observed ASVs than grass-only swards, but there was no difference in Shannon diversity. Within the grass-only treatment, season tended (*p* = 0.08) to have an effect on observed ASVs, while there was no effect within the grass-clover treatment. Within the grass-only treatment, spring had lower Shannon diversity than summer and autumn (*p* < 0.05), while summer and autumn did not differ from each other. Within the grass-clover treatment, spring tended to have lower Shannon diversity than summer (*p* = 0.08) and autumn (*p* = 0.09), with no significant difference between summer and autumn.

**Figure 2:**
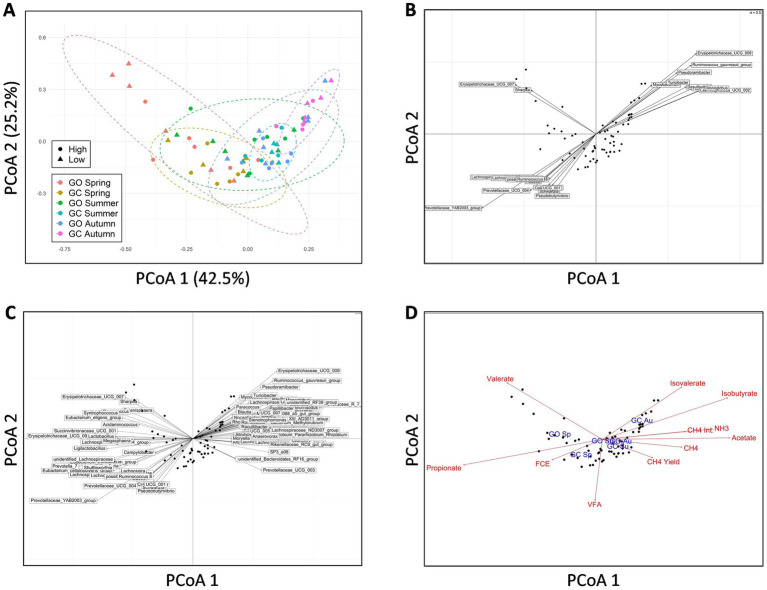
**(A)** Principal coordinates analysis (PCoA) of rumen bacterial beta the diversity in genetically divergent spring-calving dairy cows (high or low Economic Breeding Index; *n* = 11 for each genetic group) grazing grass-only swards (GO; *n* = 13) or grass-clover swards (GC; *n* = 9) in spring, summer, and autumn, showing clustering of the animals within their sward treatments during spring and autumn and separation within sward treatments across all seasons. **(B)** Co-inertia plot showing rumen bacterial genera that were significantly correlated (*p* < 0.05) with both the first and second principal axes to identify genera associated with separation across the sward treatments. **(C)** Co-inertia plot showing rumen bacterial genera that were significantly correlated (*ρ* ≥ 0.5, *p* < 0.05) with the first principal axis to identify genera associated with separation across seasons. **(D)** Correlations of animal measurements (red; CH4 = methane emissions, CH4 Int. = methane per kg of milk solids, CH4 yield = methane per kg dry matter intake, FCE = milk solids per kg dry matter intake, NH3 = rumen ammonia, VFA = rumen volatile fatty acids) and experimental factors (blue; Sp = spring, Su = summer, Au = autumn) with rumen bacterial composition at the genus level (*p* < 0.05).

Beta-diversity of the EBI groups and sward treatments across the measurement periods was investigated at the genus level using PCoA (Figure 2A). PERMANOVA showed that the EBI group had no effect on beta diversity. Grass-only and grass-clover treatments tended to separate diagonally across both the first and second principal axes in spring (*p* = 0.052) and also separated diagonally across both axes in the opposite direction in autumn (*p* < 0.01). The separation in spring was primarily associated with two divergent values within the grass-only treatment. Figure 2B shows genera associated with these separations based on their Spearman correlations with both the first and second principal axes (*p <* 0.05). A total of two genera of the *Erysipelotrichaceae* family were negatively correlated with the first axis and positively correlated with the second axis, while 11 genera were negatively correlated with both axes, five of which belonged to the *Lachnospiraceae* family. The *Prevotellaceae YAB2003 group* showed the strongest negative correlation with both axes (*ρ* = −0.80 and −0.52 for the first and second axes, respectively). In total, nine genera were positively correlated with both axes, two of which were members of the *Lachnospiraceae* family which showed the strongest correlations. PERMANOVA also showed separation within each sward treatment across the seasons, primarily along the first principal axis (*p* < 0.001). Figure 2C shows the genera that were Spearman correlated (*ρ* > 0.5, *p* < 0.05) with the first axis only. A total of 16 genera were negatively correlated with the first principal axis, with *Prevotella 7, Erysipelotrichaceae UCG 002,* and the *Prevotellaceae YAB2003 group* showing the strongest correlations (*ρ* = −0.95, −0.85, and −0.80, respectively). A total of 33 genera were positively correlated with the first principal axis, with the *Christensenellaceae R7 group*, the *Oscillospiraceae NK4A214 group*, *Mogibacterium*, *Flexilinea,* and *Eggerthellaceae DNF00809* showing the strongest correlations (*ρ* = 0.95, 0.91, 0.86, 0.86, and 0.80, respectively). Figure 2D shows the animal measurements and experimental factors that correlated (*p* < 0.05) with the rumen bacterial community. Methane, CH_4_ per kg of milk solids, CH_4_ yield, rumen ammonia, acetate, and isobutyrate were positively correlated with the first principal axis toward autumn, while feed efficiency (milk solids per kg DMI) and propionate were negatively correlated with the first principal axis toward spring. Valerate was associated with two divergent values within the grass-only treatment in spring. Isobutyrate was associated with the grass-clover treatment in autumn. Of the experimental factors tested, only season and treatment were significant (*p* < 0.05), and their positions matched those presented in Figure 2A.

The analysis of the microbiome composition found that no genera were differentially abundant between the two EBI groups. A total of 21 genera were differentially abundant between the sward treatments, although only four genera were abundant in the rumen (>1%; Table 2). *Erysipelotrichaceae UCG 002* and *Prevotella 7* were reduced, while the *Christensenellaceae R7 group* and *Oscillospiraceae NK4A214 group* were increased in the grass-clover treatment compared to the grass-only treatment (*p* < 0.05). Within individual measurement periods, there were no differences between the sward treatments. Compared with spring, 54 and 55 genera were differentially abundant in summer and autumn, respectively (*p* < 0.05; Supplementary Table S1). In agreement with the PCoA, *Prevotella 7*, *Erysipelotrichaceae UCG 002,* and the *Prevotellaceae YAB2003 group* were reduced, while the *Christensenellaceae R7 group*, *Oscillospiraceae NK4A214 group*, Olsenella, and *Butyrivibrio* were increased in both summer and autumn compared to spring. In total, there were 12 and 14 differentially abundant genera with relative abundances >1% in summer and autumn, respectively, compared with spring (Table 2). Autumn had 24 differentially abundant genera compared to summer. Among the most abundant (>1%), *Kandleria* and the *Eubacterium cellulosolvens group* decreased, while *Succiniclasticum* increased.

**Table 2:** The effect of sward type (T), dairy cow genotype (G), and season (S) on the differential abundance of the most abundant bacterial genera (>1%) in the rumen fluid of the dairy cows.

Genus	T		G		S					
	GC – GO		Low EBI – High EBI		Summer – Spring		Autumn – Spring		Autumn – Summer	
	*W*	*p*-value	*W*	*p*-value	*W*	*p*-value	*W*	*p*-value	*W*	*p-*value
*Prevotella*	0.187	0.99	−1.901	0.57	−0.067	1.00	−1.717	1.00	−1.616	1.00
*Olsenella*	0.464	0.83	−0.093	1.00	4.943	<0.001	4.763	<0.001	0.329	1.00
*Christensenellaceae R 7 group*	2.641	<0.05	0.518	0.98	3.784	<0.001	5.883	<0.001	1.990	0.09
*Kandleria*	−2.105	0.11	0.635	0.96	0.189	1.00	−5.921	<0.001	−5.194	<0.001
*NK4A214 group*	3.217	<0.05	−0.259	1.00	5.033	<0.001	6.340	<0.001	1.486	0.25
*Erysipelotrichaceae UCG 002*	−5.232	<0.001	1.128	0.78	−4.770	<0.001	−4.647	<0.001	0.646	0.92
*Prevotella 7*	−4.315	<0.001	0.458	1.00	−5.366	<0.001	−7.287	<0.001	−1.662	0.18
*Prevotellaceae UCG 001*	−1.087	0.46	−0.594	0.96	0.437	1.00	−1.225	1.00	−1.501	1.00
*Succiniclasticum*	2.131	0.11	−1.074	0.78	−2.704	<0.05	1.101	0.49	3.689	<0.01
*Butyrivibrio*	0.760	0.62	0.055	1.00	3.695	<0.01	0.785	0.77	−2.167	0.09
*Lachnospiraceae NK3A20 group*	1.138	0.44	0.600	0.96	0.021	1.00	−0.876	1.00	−0.752	1.00
*Ruminococcus*	0.746	0.63	0.608	0.96	−2.975	<0.05	−4.468	0.00	−1.716	0.16
*Acetitomaculum*	1.974	0.14	1.009	0.81	1.414	0.37	2.739	<0.05	1.275	0.37
*Eubacterium cellulosolvens group*	−2.377	0.07	0.967	0.81	−2.080	0.07	−5.342	<0.001	−3.059	<0.01
*Rikenellaceae RC9 gut group*	0.794	0.62	−1.650	0.57	4.009	<0.001	2.685	<0.05	−0.505	1.00
*Ruminococcus gauvreauii group*	0.157	1.00	1.276	0.71	0.864	0.69	3.133	<0.05	2.157	0.09

The results are expressed as the effect size (W) for the grass-clover (GC) treatment compared to the grass-only (GO) treatment, low Economic Breeding Index (EBI) compared to high EBI, and pairwise comparisons between spring, summer, and autumn.

### 3.2 Rumen archaeal community

**Figure 3:**
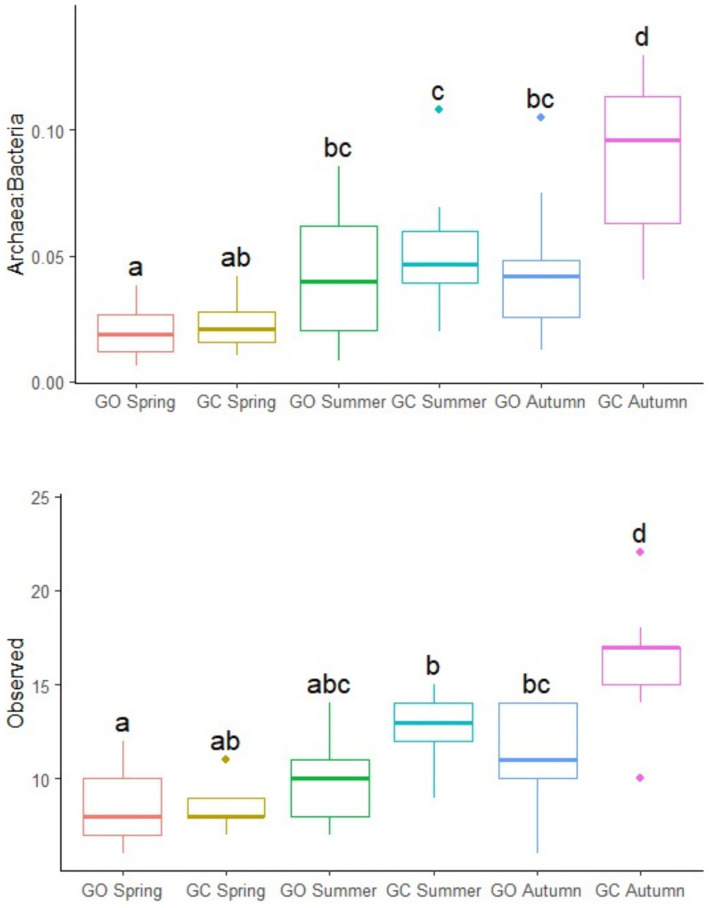
The ratio of archaea to bacteria and observed archaeal ASVs of the dairy cows grazing grass-only swards (GO; *n* = 13) and grass-clover swards (GC; *n* = 9) in spring, summer, and autumn. ^a,b,c,d^Plots not sharing a common superscript have significantly different least square means (*p <* 0.05; Tukey–Kramer adjusted).

The effects of EBI group, sward treatment, and measurement period on the rumen archaeal population are presented in Table 3. There was no effect of the EBI group on the archaeal community. The ratio of archaea to bacteria was greatest (*p* < 0.01) in the grass-clover treatment, although this was only observed in autumn (*p* < 0.001; Figure 3). The ratio of archaea to bacteria was lowest (*p* < 0.001) in spring, intermediate in summer, and greatest in autumn. Observed archaeal ASVs were greater (*p* < 0.01) in the grass-clover treatment than in the grass-only treatment. There was also an interaction with the measurement period, where grass-only and grass-clover treatments were only different in autumn (*p* < 0.001; Figure 3). Observed archaeal ASVs also increased (*p* < 0.001) from spring to summer to autumn across the sward treatments. Archaeal Shannon diversity tended to be greater (*p* < 0.1) in the grass-clover treatment than in the grass-only treatment. Shannon diversity was reduced in spring (*p* < 0.001) compared to both summer and autumn, but summer and autumn did not differ from each other. Neither the sward treatment nor the measurement period had any effect on the relative abundance of archaeal genera. Across all samples, *Methanobrevibacter* and *Methanosphaera* accounted for 91.48 and 8.51% of archaea, respectively. The relative abundance of the *Methanobrevibacter* SGMT clade was not different between the swards, but the RO clade was slightly greater (*p* < 0.05) in the grass-only treatment compared to the grass-clover treatment due to an increase in spring. The SGMT clade was reduced (*p* < 0.001) in spring compared to summer and autumn, which did not differ from each other. The RO clade was increased (*p* < 0.001) in spring compared to summer and autumn, while autumn tended to be lower than summer (*p* < 0.1).

**Table 3:** The effect of sward type (T), dairy cow genotype (G), and season (S) on the rumen archaeal community.

Item	T		SE	G		SE	S			SE	*p*-value			
	GO	GC		High	Low		Spring	Summer	Autumn		T^1^	G	S	T × S
Archaea:Bacteria	0.036	0.055	0.0547	0.044	0.046	0.0054	0.021^a^	0.047^b^	0.067^c^	0.0673	<0.05	0.81	<0.001	<0.001
Alpha diversity														
Observed ASVs	10.01	12.43	0.607	10.99	11.45	0.558	8.51^a^	11.38^b^	13.77^c^	0.500	<0.01	0.56	<0.001	<0.001
Shannon	1.83	1.92	0.039	1.88	1.87	0.036	1.74^a^	1.91^b^	1.98^b^	0.034	0.11	0.89	<0.001	0.52
Genus (%)														
*Methanobrevibacter*	92.09	90.87	0.787	91.25	91.71	0.724	91.09	91.54	91.83	0.793	0.24	0.65	0.78	0.27
*Methanosphaera*	7.90	9.11	0.789	8.74	8.27	0.725	8.91	8.44	8.17	0.792	0.25	0.65	0.78	0.28
Clade (%)														
SGMT	62.64	68.52	2.859	66.80	64.36	2.629	52.17^a^	69.68^b^	74.89^b^	2.403	0.12	0.51	<0.001	0.09
RO	27.55	20.39	2.599	22.39	25.55	2.390	37.89^a^	19.70^b^	14.32^b^	2.232	<0.05	0.35	<0.001	<0.05

^1^GO = grass-only, GC = grass-clover. ^a,b,c^Values in the same row of S not sharing a common superscript are significantly different (*p* < 0.05; Tukey–Kramer adjusted). SE, standard error of the mean.

### 3.3 Relationship between rumen bacteria and enteric CH_4_ emissions, animal performance, and rumen VFAs

Partial correlations between the most relatively abundant rumen bacterial genera (>1%) with enteric emissions, animal performance, and rumen VFA proportions are shown in Tables 4, 5. The remaining genera are shown in Supplementary Table S2. Methane production (218–446 g/d) was positively correlated (*p* < 0.05) with *Acetitomaculum*, the *Christensenellaceae R7 group,* and *Olsenella* and negatively correlated with *Prevotella 7*. Methane yield (11.8–24.5 g/kg) was positively correlated (*p* < 0.05) with *Acetitomaculum,* and the ratio of milk solids to DMI was positively correlated with *Acetitomaculum* and the *Rikenellaceae RC9 gut group*. Total VFA concentration (116–233 mmoL/L) was positively correlated (*p* < 0.05) with *Prevotella* and negatively correlated with *Acetitomaculum*, *Erysipelotrichaceae UCG 002,* and the *Lachnospiraceae NK3A20 group*. Acetate (64.3–77.1%) was positively correlated (*p* < 0.05) with *Butyrivibrio*, the *Eubacterium cellulosolvens group, the Lachnospiraceae NK3A20 group*, *Prevotellaceae UCG 001*, the *Rikenellaceae RC9 gut group*, *Ruminococcus,* and *Succiniclasticum* and negatively correlated with *Erysipelotrichaceae UCG 002* and *Prevotella 7*. Propionate (12.6–23.8%) had positive relationships (*p* < 0.05) with *Erysipelotrichaceae UCG 002* and *Prevotella 7* and negative relationships with *Acetitomaculum, Butyrivibrio, the Christensenellaceae R7 group, the Lachnospiraceae NK3A20 group, the Oscillospiraceae NK4A214 group, Olsenella, the Rikenellaceae RC9 gut group,* and the *Ruminococcus gauvreauii group*. Butyrate (5.6–10.1%) had a positive correlation (*p* < 0.05) with *Prevotella* and negative relationships with *Butyrivibrio*, the *Eubacterium cellulosolvens group*, the *Lachnospiraceae NK3A20 group,* and *Ruminococcus*. The correlations between the ratio of acetate to propionate (2.7–6.0 mmoL/L) and bacterial genera were the inverse of those of propionate (*p* < 0.05).

**Table 4:** Partial correlations between rumen bacterial genera and enteric CH_4_ emissions and animal performance^1^.

	CH_4_	CH_4_/MS	CH_4_/DMI	MS	DMI	MS/DMI
*Acetitomaculum*	0.32**	0.09	0.28*	0.2	0.01	0.24*
*Butyrivibrio*	0.19	−0.09	0.03	0.22†	0.13	0.14
*Christensenellaceae R7 group*	0.29*	0.2	0.22†	0.13	0.07	0.09
*Erysipelotrichaceae UCG 002*	−0.23†	0.09	−0.12	−0.2	−0.16	−0.11
*Eubacterium cellulosolvens group*	−0.07	−0.03	−0.13	−0.13	−0.01	−0.09
*Kandleria*	−0.09	0.05	−0.05	−0.22†	−0.11	−0.14
*Lachnospiraceae NK3A20 group*	0.16	−0.02	0.13	0.07	−0.09	0.18
*Oscillospiraceae NK4A214 group*	0.13	−0.04	0.09	0.15	0.01	0.15
*Olsenella*	0.25*	0.08	0.19	0.16	0.02	0.08
*Prevotella*	0.21†	0.04	0.14	0.16	0.14	0.06
*Prevotella 7*	−0.26*	0.01	−0.18	−0.22†	−0.11	−0.09
*Prevotellaceae UCG 001*	0.21†	0.08	0.19	0.08	0.01	0.05
*Rikenellaceae RC9 gut group*	0.04	−0.15	0.08	0.16	−0.11	0.25*
*Ruminococcus*	−0.08	−0.19	−0.15	0	0.03	0.04
*Ruminococcus gauvreauii group*	0.14	0.17	0.13	0.04	0.08	−0.05
*Succiniclasticum*	−0.01	0.06	0.09	−0.13	−0.11	−0.04

Significant relationships are indicated by ‘*’ (*p* < 0.05), ‘**’ (*p* < 0.01), and ‘***’ (*p* < 0.001), and tendencies are indicated by ‘†’ (*P* < 0.1).

MS, milk solids (fat + protein); DMI, dry matter intake.

**Table 5:** Partial correlations between rumen bacterial genera and rumen volatile fatty acids (VFA)^1^.

	Total VFA	Acetate	Propionate	Butyrate	A:P
*Acetitomaculum*	−0.28*	0.13	−0.47***	0.09	0.44***
*Butyrivibrio*	−0.22†	0.62***	−0.35**	−0.47***	0.37**
*Christensenellaceae R7 group*	−0.19	0.17	−0.63***	0.19	0.64***
*Erysipelotrichaceae UCG 002*	−0.26*	−0.46***	0.34**	−0.09	−0.27*
*Eubacterium cellulosolvens group*	−0.12	0.25*	0.15	−0.47***	−0.11
*Kandleria*	−0.12	0.01	0.20	−0.21†	−0.18
*Lachnospiraceae NK3A20 group*	−0.48***	0.28*	−0.32**	−0.34**	0.39***
*Oscillospiraceae NK4A214 group*	−0.17	0.02	−0.38**	0.14	0.41***
*Olsenella*	−0.11	0.02	−0.26*	0.16	0.22†
*Prevotella*	0.66***	0.13	0.09	0.36**	−0.18
*Prevotella 7*	−0.14	−0.41***	0.52***	−0.11	−0.42***
*Prevotellaceae UCG 001*	0.19	0.34**	−0.23†	0.07	0.23†
*Rikenellaceae RC9 gut group*	−0.11	0.28*	−0.33**	−0.09	0.36**
*Ruminococcus*	−0.05	0.25*	0.07	−0.30*	−0.04
*Ruminococcus gauvreauii group*	−0.13	0.19	−0.44***	0.08	0.44***
*Succiniclasticum*	−0.01	0.25*	−0.02	−0.18	0.00

Significant relationships are indicated by ‘*’ (*p* < 0.05), ‘**’ (*p* < 0.01), and ‘***’ (*p* < 0.001), and tendencies are indicated by ‘†’ (*p* < 0.1).

A: P, acetate to propionate ratio.

### 3.4 Relationship between rumen archaea and enteric CH_4_ emissions, animal performance, and rumen VFAs

The partial correlations of the ratio of archaea to bacteria, archaeal genera, and *Methanobrevibacter* clades with enteric CH_4_ emissions, animal performance traits, and rumen VFA proportions are shown in Tables 6, 7. The ratio of archaea to bacteria had a positive relationship with CH_4_ yield and CH_4_ per kg of MS (*p* < 0.05), as well as a positive tendency with CH_4_ production (*p* < 0.1). At the genus level, *Methanobrevibacter* abundance had a positive relationship with CH_4_ production and the ratio of acetate to propionate and a negative relationship with propionate (*p* < 0.05). *Methanosphaera* abundance had opposite relationships with *Methanobrevibacter* (*p* < 0.05). The SGMT clade had a positive relationship with CH_4_ production, DMI, total VFA concentration, and butyrate (*p* < 0.05). The RO clade tended to have a negative relationship with CH_4_ production and butyrate (*p* < 0.1) and had a negative relationship with DMI and total VFA concentration (*p* < 0.05).

**Table 6:** Partial correlations between rumen archaea and enteric CH_4_ emissions and animal performance^1^.

	CH_4_	CH_4_/MS	CH_4_/DMI	MS	DMI	MS/DMI
Archaea:Bacteria	0.24†	0.26*	0.31*	−0.03	−0.11	0.08
*Genus*						
*Methanobrevibacter*	0.29*	0.04	0.19	0.18	0.09	0.14
*Methanosphaera*	−0.29*	−0.04	−0.19	−0.18	−0.09	−0.14
*Clade*						
SGMT	0.28*	−0.04	0.03	0.24†	0.33**	0.02
RO	−0.22†	0.04	0.01	−0.20	−0.32**	0.01

Significant relationships are indicated by ‘*’ (*p* < 0.05), ‘**’ (*p* < 0.01), and ‘***’ (*p* < 0.001), and tendencies are indicated by ‘†’ (*p* < 0.1).

MS, milk solids (fat + protein); DMI, dry matter intake; SGMT, *Methanobrevibacter smithii*, *gottschalkii, millerae*, and *thaueri*; RO, *Methanobrevibacter ruminantium* and *olleyae.*

**Table 7:** Partial correlations between rumen archaea and rumen volatile fatty acids (VFA)^1^.

	Total VFA	Acetate	Propionate	Butyrate	A:P
Archaea:Bacteria	−0.23†	−0.01	−0.46***	0.19	0.45***
*Genus*					
*Methanobrevibacter*	−0.16	−0.16	−0.34**	0.21†	0.29*
*Methanosphaera*	0.16	0.16	0.34**	−0.21†	−0.29*
*Clade*					
SGMT	0.46***	0.09	−0.22†	0.25*	0.09
RO	−0.55***	−0.05	0.16	−0.24†	−0.03

Significant relationships are indicated by ‘*’ (*p* < 0.05), ‘**’ (*p* < 0.01), and ‘***’ (*p* < 0.001), and tendencies are indicated by ‘†’ (*p* < 0.1).

A: P, acetate to propionate ratio; SGMT, *Methanobrevibacter smithii, gottschalkii, millerae*, and *thaueri*; RO, *Methanobrevibacter ruminantium* and *olleyae.*

## 4 Discussion

The current study examined ruminal bacteria and archaea populations of genetically divergent dairy cows grazing grass-only or grass-clover swards at different stages of the grazing season. Similar to previous findings in autumn, there was no difference in bacterial Shannon diversity between the sward treatments across all three measurement periods (Smith et al., 2020). In addition, the abundances of the main rumen bacterial genera were similar between the cows grazing the two sward treatments in the current study. *Prevotella* dominated the microbiome of the cows grazing both sward types, which is consistently reported across a wide variety of diets (Henderson et al., 2015; Lyons et al., 2018; Zhu et al., 2021). As such, *Prevotella* is often labeled a generalist, owing to its ability to degrade a wide variety of plant cell wall and storage polysaccharides (Betancur-Murillo et al., 2023). However, there was a difference in *Erysipelotrichaceae UCG002* between the two swards, although this genus was primarily associated with two divergent values in the grass-only treatment in spring. The co-inertia plots also showed that these values were associated with *Sharpea* and *Erysipelotrichaceae UCG007*. *Sharpea* and other members of the *Erysipelotrichaceae* family are associated with lactic acid production (Doyle et al., 2019; Chen et al., 2021; Zhou et al., 2024); therefore, these animals may have been experiencing rumen dysbiosis due to low rumen pH at the time of sampling (Monteiro and Faciola, 2020). Low rumen pH is common among spring-calving dairy cows grazing highly digestible spring pastures, although proliferation of lactic acid bacteria is not (Kolver and de Veth, 2002; O’Grady et al., 2008). However, rumen pH was not measured in the current study; therefore, no conclusions can be drawn.

Similar to Smith et al. (2020), we observed an overall difference in microbial community composition between the two sward treatments in autumn. This was likely due to the increased clover proportions (50.2%) during this measurement period, providing a greater diversity of substrates available in the rumen, such as pectin and its subsequent metabolites (Ulyatt, 1971; Van Soest, 1994). The digestion of pectin in animals grazing grass-clover swards has been previously associated with methanol production, a substrate for methylotrophic methanogenesis (Kelly et al., 2019). Previous studies have linked pectin-derived methanol with a greater abundance of the methylotrophic methanogen, *Methanosphaera*, in animals consuming grass-clover swards (Smith et al., 2020; Woodmartin et al., 2024). No such relationship was observed in the current study. Generally, feeds with lower neutral detergent fiber (NDF) levels, such as the grass-clover sward in the current study, are associated with reduced CH_4_ yield, as they are associated with lower rumen H_2_ levels (Van Soest, 1994; Janssen, 2010). However, this was not observed in our previously reported data (Dwan et al., 2025). We previously postulated that pectin digestion may also contribute to hydrogenotrophic methanogenesis in the rumen (Dwan et al., 2025), as it has been reported to produce acetate and formate when digested (Dušková and Marounek, 2001). Formate and H_2_ associated with acetate are utilized by *Methanobrevibacter* in hydrogenotrophic methanogenesis (Tapio et al., 2017). This may explain why the grass-clover sward does not have the lower CH_4_ yield that would be expected of a feed with lower NDF levels. In general, archaeal abundance was greater in the grass-clover sward during autumn in the current study. It is unclear why this occurred and why it was not associated with an increase in CH_4_ yield compared to the grass-only sward (Dwan et al., 2025). It could theoretically be a result of differences in digestive kinetics between the two swards (Wang et al., 2016). Although the digestive products of pectin are similar to those of NDF, its digestion rate is much faster and may contribute to an increased digestion rate in cows grazing grass-clover swards (Steg et al., 1994; Van Soest, 1994). A limitation of the current study is that the rumen samples were collected from all cows at one time point only (01:30 p.m.) during each measurement period. The spike in methanogen abundance observed in the grass-clover treatment during autumn may be a result of more rapid digestion of their morning grazing compared to the grass-only treatment and may not be representative of methanogen abundance across the entire day (Wang et al., 2016; Hao et al., 2024). This is important considering that cows have a partial preference for clover over PRG, which is reported to be greater in the morning compared to later in the day (Rutter et al., 2004). Future investigation is required into the rumen digestive kinetics of cows grazing grass-clover swards and their associations with the rumen microbial community.

The composition of the rumen microbiome has a major influence on the end products of ruminal fermentation (Russell and Hespell, 1981). Roehe et al. (2016) noted a greater abundance of methanogenic genes in the rumen metatranscriptome of cattle phenotypically high for CH_4_ production. These authors also suggested that archaeal abundance is under host genetic control and is an accurate predictor of CH_4_ output. Therefore, the fact that we observed no differences in the rumen microbial communities of the two dairy cow genotypes in the current study could be expected based on their similar rumen fermentation characteristics and enteric CH_4_ emissions (Dwan et al., 2025). These results suggest that the improved feed efficiency and greater milk constituents of the high EBI group are largely a result of physiological, as opposed to digestive, mechanisms, although a greater sample size could potentially reveal some subtle differences in the microbiome between the two genotypes. Nonetheless, our observation is similar to the findings of studies with comparable sample sizes investigating cows divergent in residual feed intake, where no differences in the rumen microbial community were observed (Rius et al., 2012; Noel et al., 2019). Despite the similar microbial populations in the current study, future research should investigate the rumen metagenome of genetically divergent dairy cows to identify potential associations between host genetics, phenotypic performance, and microbial gene content, which can differ significantly among strains of the same microbial species (De Filippis et al., 2019).

A novel aspect of the current study is its investigation of the rumen microbial community of spring-calving dairy cows at different stages of the grazing season. Noel et al. (2017) reported minor seasonal fluctuations in the rumen microbiome composition of grazing dairy cows, albeit that study focused on fiber-adherent microbes and included only four subjects. The current study is important in the context of previous research that reported variation in CH_4_ yield across the grazing season, with particularly lower levels observed in spring (Robertson and Waghorn, 2002; Lahart et al., 2024). We acknowledge that the effect of season cannot be separated from lactation stage within such systems; however, previous studies on consistent total mixed ration (TMR) diets have reported only a minor effect of lactation stage on CH_4_ yield and the rumen microbial community (Bainbridge et al., 2016; Lyons et al., 2018; Zhu et al., 2021; Fresco et al., 2023). Across various analyses in the current study, we observed significant differences in both rumen bacterial and archaeal communities across seasons. With the exception of grass-clover swards in autumn, the ratios of archaea to bacteria across the three seasons in the current study align with our CH_4_ yield data (Dwan et al., 2025) and those reported by previous studies (Robertson and Waghorn, 2002; Lahart et al., 2024). Our results suggest that differences in archaeal abundance across seasons may be driven by shifts in rumen VFA proportions.

Changes in sward quality across the grazing season are associated with a shift in rumen VFA proportions, from greater propionate levels in spring to greater acetate levels in summer and autumn (McAuliffe et al., 2022). Previous data indicate that methanogens are negatively associated with propionate in the rumen (Stepanchenko et al., 2023), which is in agreement with the results obtained from the current study. There are a number of probable reasons for this association: firstly, propionate production at the expense of acetate and butyrate production reduces H_2_ production and, subsequently, availability for methanogen growth (Janssen, 2010; Wang et al., 2014). Secondly, propionate production is generally associated with more rapidly digestible feeds, which reduce rumen pH and increase rumen passage rates, both of which are detrimental for methanogen growth (Van Soest, 1994; Van Kessel and Russell, 1996; Janssen, 2010). Furthermore, the H_2−_utilizing bacterial genus *Acetitomaculum* (Greening and Leedle, 1989) showed a similar trend in abundance to the archaea in the current study, which further supports the idea that their abundances are related to rumen H_2_ concentrations across seasons. Elevated propionate levels are driven by higher sward water-soluble carbohydrate levels as a result of lower NDF content, which is indicative of high-quality spring pasture (Kolver and de Veth, 2002; Taweel et al., 2005). This shift in sward quality was associated with a shift in the rumen bacterial community, particularly between spring and the other two seasons, in the current study. The two bacterial genera with the greatest reduction in abundance from spring to the later seasons were *Erysipelotrichaceae UCG002* and *Prevotella* 7. The *Erysipelotrichaceae* family has been shown to be highly abundant in grazing dairy cows in spring, a pattern associated with lower CH_4_ yield compared to a TMR diet (de Menezes et al., 2011; O’Neill et al., 2011). Species in the *Prevotella 7* genus are predicted to digest non-structural carbohydrates, such as starch, and produce propionate (Hitch et al., 2022), which is in line with the partial correlations observed in the current study. Furthermore, the bacterial genera with the greatest increase in abundance in summer and autumn compared to spring in the current study—*Olsnella*, the *Christensenellaceae R7 group*, the *Oscillospiraceae NK4A214 group*, the *Rikenellaceae RC9 group,* and *Butyrivibrio*—have been associated with forage digestion and/or acetate production (Mackie Roderick et al., 2003; Kraatz et al., 2011; Morotomi et al., 2012; Asma et al., 2013; Palevich et al., 2019). This was further substantiated by the partial correlations observed in the current study, where each genus was negatively correlated with propionate and positively correlated with either acetate or butyrate, both of which would theoretically enhance H_2_ availability for methanogenesis (Janssen, 2010).

Our results show that the core rumen microbiome of grazing dairy cows is robust to differences in sward composition, facilitating similar levels of animal performance across grass-only and grass-clover swards (Enriquez-Hidalgo et al., 2014; Dwan et al., 2025). In addition, based on our data, genetic selection using the EBI has no effect on rumen bacterial and archaeal populations. The results from the current study also indicate that archaeal abundance has the strongest association with the CH_4_ yield of any microbiota parameter tested and that there is a significant change in their abundance across the grazing season, irrespective of sward treatment, aligning with seasonal variations in CH_4_ yield. These changes seem to be largely driven by changes in sward quality across the grazing season, shifting the composition of the rumen bacterial community, which alters rumen fermentation characteristics. Future investigations into the rumen metagenome and the expression of genes related to hydrogen production and methanogenesis would further improve our understanding of this relationship.

## Data Availability

The datasets presented in this study are publicly available. This data can be found at: https://www.ncbi.nlm.nih.gov/, accession number PRJNA1273030.

## Footnotes

^1^ https://www.arb-silva.de/

^2^ https://zenodo.org/record/2541239
