# Supplementary material for: Evaluating the rumen microbial community of genetically divergent spring-calving dairy cows grazing grass-only or grass-clover swards at different stages of the grazing season
Source: Front Microbiol. 2025 Nov 6;16:1642486. doi: 10.3389/fmicb.2025.1642486 (PMC12633751; doi:10.3389/fmicb.2025.1642486)
Supplement: Supplementary file 2 [file Supplementary_file_1.docx]

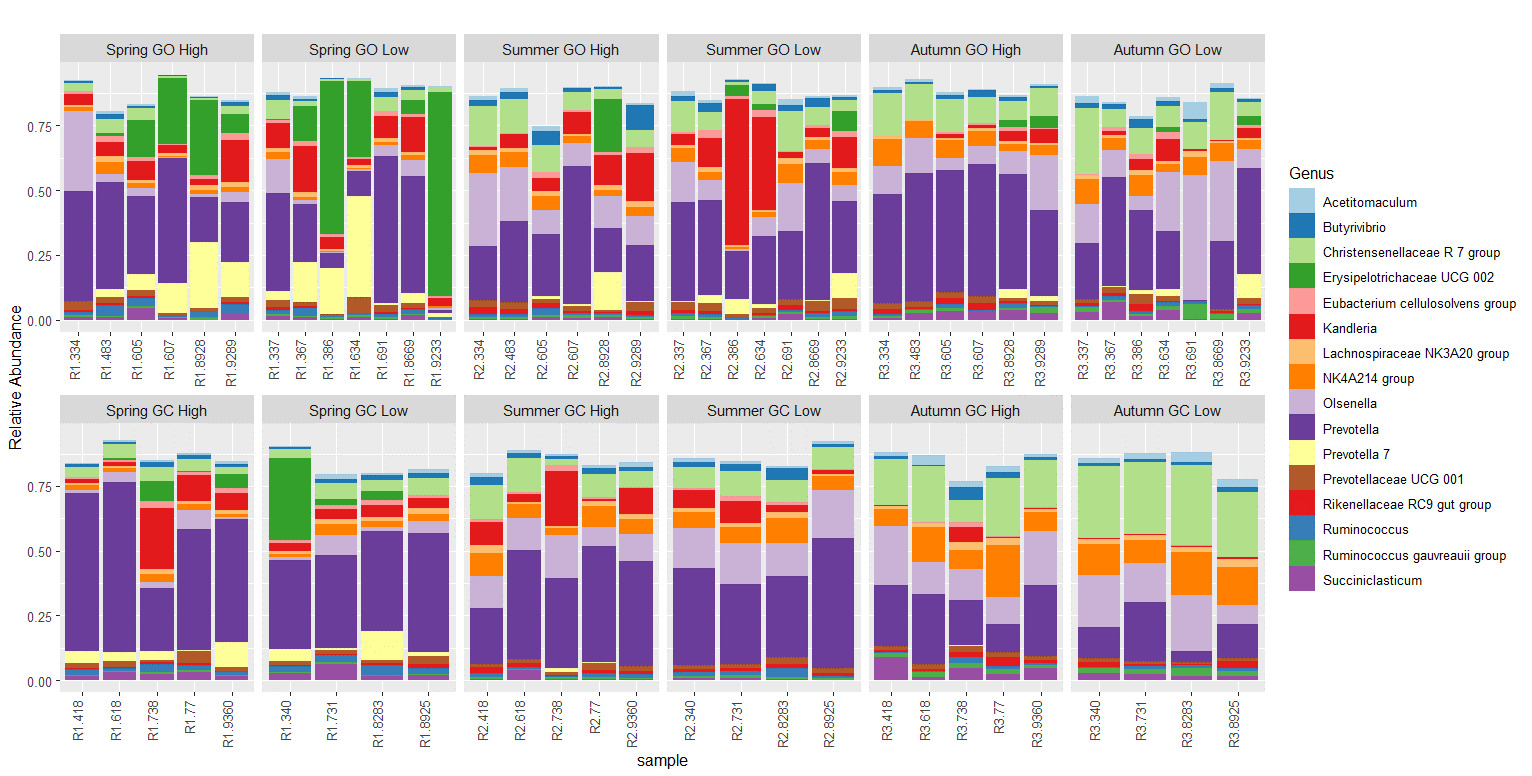


**Supplementary Figure 1.** The relative abundance of the most prevalent (> 1%) bacterial genera in each rumen fluid sample from genetically divergent dairy cows (‘High’ or ‘Low’) grazing grass-only (GO) and grass-clover (GC) in spring, summer and autumn.
